# Utility of Bone Marrow Examination in Pediatric Age Group: Experience of a Tertiary Healthcare Centre in India

**DOI:** 10.7759/cureus.27056

**Published:** 2022-07-20

**Authors:** Meenakshi Balasubramanian, Niyati N Sangoi

**Affiliations:** 1 Pathology, Topiwala National Medical College and Bai Yamunabai Laxman Nair Charitable Hospital, Mumbai, IND

**Keywords:** anemia, acute leukemia, pediatric, bone marrow biopsy, bone marrow aspiration

## Abstract

Introduction

Bone marrow is involved in various haematological and non-haematological conditions in the paediatric age group. To understand and analyse the underlying etiology, bone marrow examination, including both bone marrow aspiration and trephine biopsy, forms an important diagnostic tool. It helps to differentiate proliferative disorders of the myeloid and lymphoid series, find out their prognosis based on severity, and assess their status before therapy is started. It also aids in staging and in the detection of infiltration of the marrow by foreign cells. The spectrum of disorders affecting the marrow in children ranges from common disorders like nutritional deficiency anaemia to serious conditions like leukaemia. Their line of management, severity, and prognosis differ from the bone marrow lesions found in adults. The present study was done to demonstrate the utility of bone marrow examination in the paediatric age group, to demonstrate its contribution to the final diagnosis of the patient, and to list and study the various lesions encountered in this age group in a tertiary care teaching hospital and for better understanding of clinicopathological features and microscopy, suggesting variation with or convergence with literature.

Material and methods

This was an observational study with a combination of retrospective and prospective studies, carried out over a period of six years, from January 2014 to December 2019. All bone marrow aspiration and biopsy samples received in the surgical pathology department of our tertiary healthcare centre during the study period of patients between zero and 18 years of age were included. Samples obtained at autopsy and samples where the tissue was diluted or the marrow was inadequate were excluded from the study. Detailed histomorphology was studied in all cases, and the data obtained was recorded in an excel sheet. The descriptive statistics were expressed in terms of numbers and percentages. The study was ethically approved by the Ethics and Research Committee of our hospital before the commencement of the study.

Results

The maximum number of marrows were performed in the age group of 12-18 years, followed by six to 12 years. The most common indication for performing bone marrow examination was ‘evaluation of cytopenias’, followed by ‘suspected/to rule out haematological malignancy’. A male predominance was found. Overall and amongst neoplastic lesions, acute leukaemia was the commonest diagnosis in the bone marrow. Infections were the second most common lesion overall and the commonest non-neoplastic lesion encountered.

Conclusion

The spectrum of lesions requiring bone marrow examination in the paediatric age group is of varied etiologies. Bone marrow examination in children is useful in understanding the pathogenesis, establishing a tissue diagnosis, ruling out haematological malignancies and other causes of cytopenias, planning further lines of investigations and management, and knowing the response to therapy and prognosis.

## Introduction

Various haematological and non-haematological conditions involve the bone marrow. Thus, to understand and analyse the underlying etiology, bone marrow examination, which includes bone marrow aspiration and bone marrow trephine biopsy, forms an important diagnostic tool. Reliable information regarding the morphology and stage of maturation of blood cells is provided by bone marrow aspirate [1]. Trephine biopsy provides additional information regarding marrow cellularity, architecture, and infiltration. Bone marrow examination helps to differentiate proliferative disorders of the myeloid and lymphoid series, find out their prognosis based on severity, and assess their status before and after appropriate treatment is given. It also aids in the staging and infiltration of the marrow by foreign cells [2]. The basic indication for performing a bone marrow examination is to answer questions that were not answered by routine examination of a blood sample [3]. There have been major advances in recent years in understanding bone marrow architecture and the relationship between its tissue and cellular elements, which are of paramount importance to understand the behaviour of both neoplastic and non-neoplastic lesions. Hematological disorders are commonly seen in the paediatric age group. The spectrum ranges from common disorders like nutritional deficiency anaemia to serious conditions like leukaemia. Their line of management, severity, and prognosis differ from the bone marrow lesions found in adults. Although bone marrow examination is an invasive procedure, it can be performed easily even in cases with severe thrombocytopenia with little or no risk of bleeding [1]. The present study was conducted to study the diagnostic utility of bone marrow examination and the spectrum of lesions in bone marrow in the paediatric age group.

## Materials and methods

This was an observational study with a combination of retrospective and prospective studies, carried out over a period of 72 months. For the retrospective study, details about the patient were taken from medical case records like age, sex, chief complaints, investigations, and clinical diagnosis. Bone marrow biopsy and bone marrow aspirate slides were retrieved from the surgical histopathology section of the pathology department of our tertiary care centre and studied. If additional slides were required, the sections were taken from cut paraffin blocks, and the slides were stained and studied. For a prospective study, specimens of bone marrow in 10% formalin and stained/unstained slides of bone marrow aspirate were received in the surgical department of pathology. Bone marrow biopsy specimens were stained with routine hematoxylin and eosin. Special stains like Reticulin, Masson’s Trichrome, Zeil-Neelson, and Grocott Methenamine Silver (GMS) stain were done in required cases to confirm the histopathological diagnosis. Bone marrow aspirate slides were stained with Leishman’s stain. Special stains like Periodic Acid Schiff were done in required cases. The study was ethically approved by the ethics and research committee of our hospital, namely the Ethics Committee for Academic Research Projects (ECARP), before the commencement of the study (approval number - ECARP/2018/107). All bone marrow aspiration and bone marrow biopsy samples received during the study period from patients between zero and 18 years of age were included. Samples obtained at autopsy and samples where the tissue was diluted or the marrow was inadequate were excluded from the study. This was an observational study, and the data obtained were recorded on an Excel sheet. The descriptive statistics were expressed in terms of numbers and percentages. Tables and graphs were used to present the final data.

## Results

A total of 150 bone marrow examinations were carried out during the study period, based on the inclusion and exclusion criteria. A maximum number of cases were seen in the age group of 12-18 years (Table 1). The mean age of the study population was 9.2 years. Of these, 101 cases (67.3%) were males and 49 cases (32.7%) were females. The male to female ratio was 2.06:1. The most common indication for performing bone marrow examinations in children was the evaluation of cytopenias (42.6%), followed by suspected haematological malignancy (30%). Other indications are mentioned in Table 2.

**Table 1:** Age-wise distribution (total cases=n=150)

Age group	Number of cases	Percentage (%)
0–1 month	0	0
1 month to 1 year	9	6
1–6 years	40	26.7
6–12 years	44	29.3
12–18 years	57	38

**Table 2:** Indications to perform bone marrow examination in children (n=150)

Indication	Total	Percentage (%)
Evaluation of cytopenias	64	42.6
Suspected/to rule out haematological malignancy	45	30
To evaluate remission in malignancies involving the marrow	15	10
Pyrexia of unknown origin (PUO)	9	6
Staging	6	4
Suspected storage disorder	3	2
Miscellaneous	8	5.3

Fever was the most common presenting symptom, followed by bleeding manifestations and generalised weakness (Table 3). Pallor was the most common presenting sign, followed by splenomegaly and hepatomegaly (Table 4). The majority of cases were of ‘acute leukaemias’, constituting 27.3% (41 cases) of the total number of cases. Infections were the second most common etiology, constituting 14% (21 cases) of the total number of cases. Apart from infections, the non-malignant disorders reported in bone marrow were nutritional anaemia, aplastic anaemia, immune thrombocytopenic purpura (ITP), eosinophilia, storage disorders, suspected haemophagocytic lymphohistiocytosis (HLH), myelofibrosis secondary to rickets, and erythroid hypoplasia. A bone marrow sample of a suspected case of TAR (thrombocytopenia with absent radii) syndrome was also received. Apart from acute leukemia, the malignant disorders reported in the marrow were chronic myeloproliferative disorders (CMPD) favouring chronic myeloid leukaemia (CML), lymphoma, myelodysplastic syndrome (MDS), and juvenile myelomonocytic leukaemia (JMML). Out of the seven marrows received in patients diagnosed with lymphoma, only one showed bone marrow involvement. We also received 15 bone marrow samples as a part of the management protocol and follow-up in children with acute leukaemia. The distribution of cases based on the aetiology of bone marrow examination is mentioned in detail in Table 5.

**Table 3:** Distribution of main presenting symptoms

Symptom	Number of cases	Percentage
Fever	85	56.6%
Bleeding manifestations	28	18.6%
Generalized weakness	24	16%
Abdominal pain	14	9.3%
Breathlessness	13	8.7%

**Table 4:** Distribution of main presenting signs

Symptom	Number of cases	Percentage
Pallor	46	30.6%
Splenomegaly	36	24%
Hepatomegaly	30	20%
Lymphadenopathy	18	12%
Jaundice	12	8%

**Table 5:** Distribution of cases based on etiology ITP: immune thrombocytopenic purpura, CMPD: chronic myeloproliferative disorder, CML: chronic myeloid leukemia, HLH: hemophagocytic lymphohistiocytosis, JMML: juvenile myelomonocytic leukemia, TAR: thrombocytopenia with absent radii

Sr. No.	Diagnosis	No. of cases	Percentage (n=150)
1	Acute leukemia	41	27.3%
2	Infections	21	14%
3	Nutritional anemia	20	13.3%
4	Aplastic anemia	15	10%
5	ITP	9	6%
6	Marrow in lymphoma	7	4.6%
7	CMPD favouring CML	5	3.3%
8	Eosinophilia	4	2.7%
9	Relapse of leukemia	3	2%
10	Suspected HLH	2	1.3%
11	Storage disorder	2	1.3%
12	Myelodysplastic syndrome	2	1.3%
13	Erythroid hypoplasia	1	0.66%
14	JMML	1	0.66%
15	Myelofibrosis	1	0.66%
16	Suspected TAR syndrome	1	0.66%
17	Marrow received as a part of management protocol and follow-up in cases of acute leukemia	15	10%
	Total	150	

## Discussion

The spectrum of haematological and non-haematological disorders requiring bone marrow examination is different in the paediatric age group as compared to adults. Hence, this study was undertaken for a better understanding of clinicopathological features and microscopy, variation with or convergence with literature, and to enhance diagnostic skills. We received 150 bone marrow aspirate and/or trephine biopsy samples that satisfied the adequacy criteria. Of these, 53 cases (35.3%) were malignant and 82 cases (54.7%) were non-malignant.

We found the maximum number of cases in the age group of 12-18 years, followed by the age group of 6-12 years. This was similar to the study conducted by Patil et al. [2], where the maximum number of cases were seen in the 12-18 year age group, followed by the 5-12 year age group. In a study by Shams et al. [4], the maximum number of cases in children was between 12 and 16 years of age, similar to our study. We did not receive bone marrow samples from neonates in our study, which was also seen in the study done by Patil et al. [2]. We found male predominance with 67.3% of males and 32.7% of females and a male:female ratio of 2.06. The studies by Patil et al. [2], Majumdar et al. [1], and Fatima et al. [5] reported a male:female ratio of 1.66, 1.5, and 1.2, respectively. Our study showed a higher percentage of males as compared to the other studies, and this could be because of the variation in the duration of the study and the number of cases.

Evaluation of cytopenias was the most common indication to do a bone marrow examination in our study, comprising 42.6% of all the indications. Suspected/to rule out haematological malignancy was the second most common indication, comprising 30% of all the indications. This was comparable to the studies by Majumdar et al. [1], Pudasaini et al. [6], and Bashawri et al. [7], as all three studies reported pancytopenia and the diagnosis and management of leukaemia as the two most common indications. However, in the study done by Fatima et al. [5], the most common indications in the descending order of frequency were persistent fever, anaemia, and leukaemia. A study done by Patil et al. [2] reported anaemia as the most common indication to do a bone marrow examination, whereas, in the study done by Githang’A et al. [8], the most common indication for bone marrow examination was haematological and non-haematological malignancies.

The commonest presenting symptoms encountered in our study were fever (56.6% of cases), bleeding manifestations (18.6% of cases), and generalised weakness (16% of cases). The study done by Patil et al. [2] also showed fever and general debility as the commonest presenting symptoms, seen in 56.6% and 38.3% of cases, respectively. In their study, gastrointestinal disturbances were the third most common presenting symptoms. In contrast, studies by Shams et al. [4] and Chandra et al. [9] showed generalised weakness as the commonest presenting symptom. In our study, pallor was the most common presenting sign (30.6% of cases), followed by splenomegaly (24% of cases). This was similar to the study done by Shams et al. [4], where pallor and splenomegaly were the most common presenting signs.

**Figure 1:**
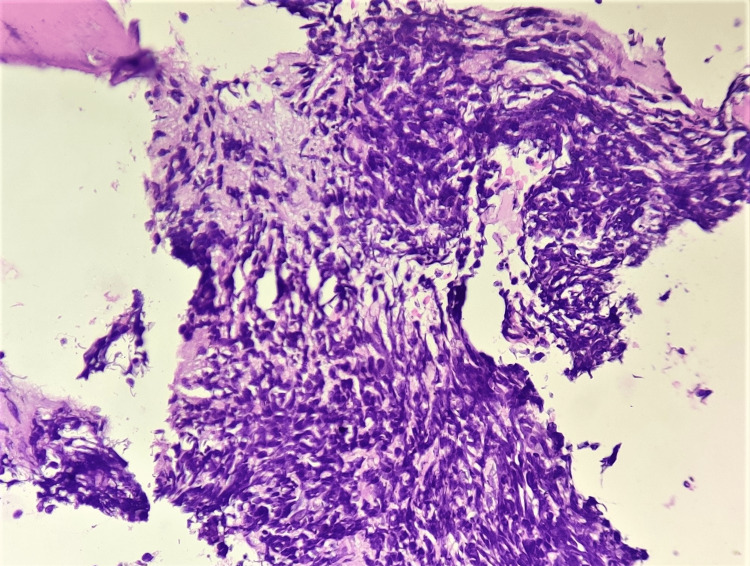
Bone marrow trephine biopsy in acute lymphoblastic leukemia Bone marrow trephine biopsy of a child diagnosed with acute lymphoblastic leukemia, showing marrow packed with blasts and extensive crushing artifact (H&E, 400×).

In our study, the commonest diagnosis of bone marrow was ‘acute leukemia'. Out of 41 cases, 28 (68.3%) were males and 13 (31.7%) were females. Thus, our study showed a significant male predominance. Studies done by Iram Ali et al. [3] and Lingayat et al. [10] showed only a slight male predominance. Follow-up with the final diagnosis and subclassification of leukaemia was obtained in 31 out of 41 cases. Of these, 25 cases (80.6%) constituted acute lymphocytic leukaemia (ALL) and 6 cases (19.4%) constituted acute myeloid leukaemia (AML). Similar results were shown in the studies done by Patil et al. [2], Iram Ali et al. [3], Githang’A et al. [8] and Rahim et al. [11], with ALL being the commonest haematological malignancy in children. However, the study done by Hasenbegvoic et al. [12] showed an equal frequency of ALL and AML. We reported a case of ‘acute panmyelosis with myelofibrosis (APMF)’ in a 16-year-old male. The patient presented with pancytopenia with the peripheral smear showing an increased number of nucleated RBCs with the presence of dyserythropoiesis. The aspirate was hypocellular and showed the presence of blasts. The hypocellularity in the aspirate was possibly because of fibrosis. Trephine biopsy of this case revealed trilineage hyperplasia with increased clustering of megakaryocytes seen in places with the presence of hypolobated and non-lobated megakaryocytes. Reticulin stain revealed grade 2 fibrosis. Follow-up revealed that immunohistochemistry was not contributing towards a definite diagnosis and the patient was treated as a case of acute myeloid leukaemia and there was improvement in symptoms. Many published articles consider APMF as a variant of myelodysplastic syndrome [13]. It is important to have knowledge about this entity since these patients need aggressive management due to the rapidly fatal course and poor outcome associated with this condition. Thus, in all cases of acute leukemia, both bone marrow aspirate and trephine biopsy should be done, as they complement each other. Figure 1 shows a bone marrow trephine biopsy of a child diagnosed with ALL, showing marrow packed with blasts and extensive crushing artefacts.

We categorised 21 cases (14% of all cases) as ‘infectious diseases’ based on an analysis of combined clinicopathological, bone marrow, and follow-up findings, thus being the second most common aetiology of our study. In the majority of the cases, the cause of the infection was non-specific. The bone marrow examination findings in the study done by Kumar et al. [14], which explained the role of bone marrow examination in cases of infection, showed erythroid hyperplasia, dyshemopoiesis, plasmacytosis, lymphocytosis, increased number of histiocytes and plasma cells, necrosis, and hemophagocytosis, which were also seen in the present study.

In the present study, only 20 cases (13.3%) were diagnosed with nutritional anemia. This is in contrast to Patil et al. [2] and Iram Ali et al. [3], which showed nutritional anaemia as the most common condition encountered in their studies. The prevalence of anaemia in children is over 70% in most parts of India and Asia [15]. Thus, the percentage of nutritional anaemia in our study is lower than expected. This may be because a majority of cases with nutritional deficiency anaemia present as isolated anaemia rather than pancytopenia and are diagnosed on peripheral smear and serum studies, thus being managed on an outpatient basis. The threshold for performing a bone marrow examination for such patients, especially in children, was high at our centre. Iron deficiency anaemia was seen in 35% of cases, dimorphic anaemia was seen in 50% of cases, and megaloblastic anaemia was seen in 15% of cases. Bone marrow aspirate was more useful as compared to trephine biopsy to appreciate the morphological features seen in megaloblastic anaemia.

**Figure 2:**
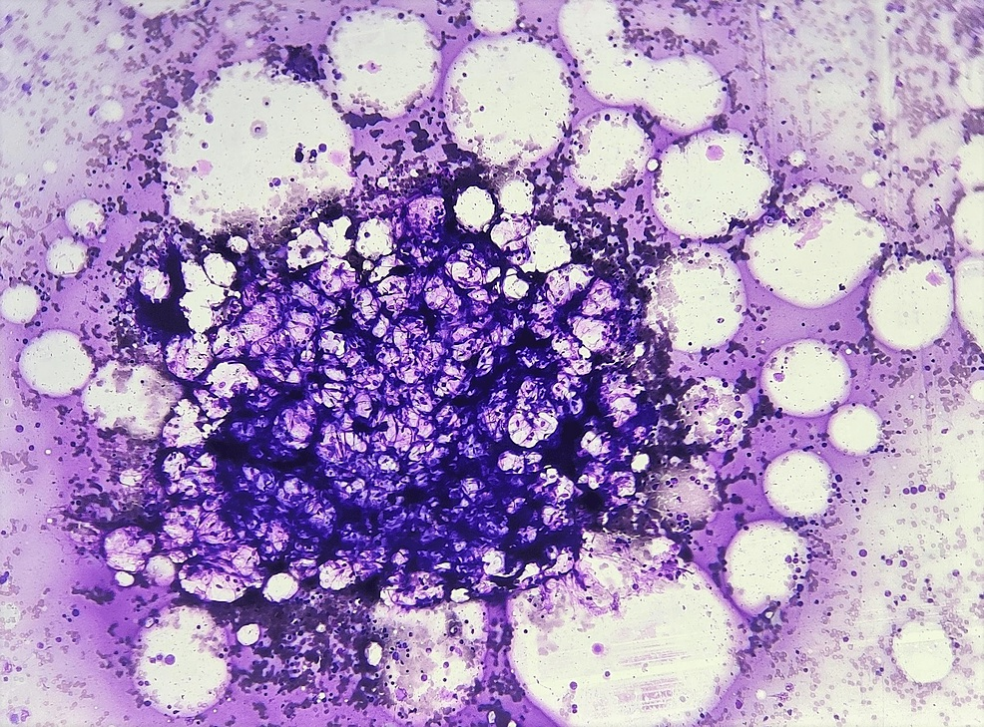
Bird's nest appearance in aplastic anemia Bone marrow aspirate showing hypoplastic fragment giving the characteristic bird’s nest appearance, seen in aplastic anemia (Leishman’s stain, 400×).

The third most common non-malignant haematological disorder found in our study was aplastic anaemia (9.3%), similar to the study done by Majumdar et al. [1]. Our study showed a male predominance and the maximum cases were seen in the age group of 12-18 years. These findings were similar to a study done by Hossain et al. [16], which included children aged zero to 19 years, with 54.6% males and 45.4% females, with the maximum number of cases seen in the age group of 11 to <16 years. A dry tap was obtained in four cases, while seven cases showed the smear to be completely diluted by peripheral blood. The rest of the cases showed a markedly hypocellular marrow with a reduction in erythroid, myeloid, and megakaryocytic series, with two cases showing a ‘bird’s nest appearance’ of the fragments (Figure 2). There was a relative increase in lymphocytes and plasma cells seen in these cases. The majority of the cases in a study done by Daniel and Byrds [17] showed hypocellular marrow with an increase in lymphocytes.

Nine cases (6%) were reported as ITP, of which four (44.4%) were males and five (55.6%) were females. In the study by Iram Ali et al. [3], ITP was the third most common haematological disorder. Their study showed a male predominance (64.7%), while a slight female predominance was seen in our study (55.6%). Unlike adults, where ITP shows a female predominance, ITP in children affects males and females equally [18]. The majority of the patients in the present study were in the age group of one to six years. Iram Ali et al. [3] reported petechiae to be the most common presenting symptom in children with ITP. Splenomegaly was not reported in any of the cases. This was similar to our study, which showed bleeding manifestations to be the most common presenting symptom and an absence of splenomegaly in all cases.

We received bone marrow samples from seven cases of lymphoma, of which four cases were Hodgkin’s lymphoma and three cases were non-Hodgkin’s lymphoma. Of these, only one case, which was a 14-year-old female diagnosed with non-Hodgkin’s lymphoma on cervical lymph node biopsy, showed bone marrow involvement. Bone marrow examination showed total replacement of marrow by the proliferation of a monotonous population of cells with an altered nuclear:cytoplasmic ratio and scant cytoplasm, resembling lymphoid cells.

In our study, we encountered five cases (3.3%) of CMPD favouring CML. However, we did not get the follow-up molecular diagnostics reports to confirm the diagnosis. CML is extremely rare below the age of one year. CML is usually seen in children older than 12 years in India [19]. This is reflected well in our study, in which the maximum cases (four out of five) were seen in the age group of 12-18 years.

We encountered three cases of ALL that relapsed during treatment. Two cases presented with fever, and two cases showed the presence of abnormal cells on a peripheral smear. A study done by Cornbleet et al. [20] concluded that bone marrow relapse was equally serious whether it occurred during treatment or after treatment had been stopped, and that most children with ALL had a single chance of cure at the time of diagnosis. Careful monitoring and follow-up should be ensured in all patients with acute leukaemia from the beginning for prevention and early diagnosis of relapse.

We received two marrow samples from patients with suspected HLH. Both were males with the ages of seven months and four years, respectively. The seven-month-old child had a fever, splenomegaly, hypertriglyceridemia, cytopenia in two cell lines (anemia and thrombocytopenia), and hemophagocytosis in the marrow, thus fulfilling five parameters required to diagnose HLH. The four-year-old child presented with fever and hepatosplenomegaly and had thrombocytopenia and neutrophilic leucocytosis on the peripheral smear. Bone marrow aspirate showed a mild increase in histiocytes with occasional histiocytes showing hemophagocytosis. However, we could not get the other laboratory parameters required to diagnose HLH as the patient was lost to follow-up. Nandhakumar et al. [21] studied 52 children who fulfilled the criteria for HLH. Of these, 13% were confirmed to have familial HLH while 87% had secondary HLH. The commonest cause of secondary HLH in their study was viral infections, with dengue being the most common. The study concluded that the initial presentation of HLH may be vague and hence, a very high clinical suspicion and structured work-up including immunologic and genetic testing is needed. Since tropical infections are a major cause of HLH in India, prompt initiation of appropriate antimicrobial treatment as indicated along with supportive therapy can be life-saving. It is important to recognise and diagnose HLH early, even if all the required criteria are not met, as the manifestations of the disease may evolve over time. The study by Ho et al. [22] states that early in the disease process, hemophagocytosis may not be clearly apparent in the bone marrow, and hence, the absence of hemophagocytosis does not rule out HLH.

Two cases (1.3%) were reported as storage disorders, one being Gaucher’s disease and the other being Neimann-Pick disease in a five-year-old female and a nine-month-old male, respectively. Both cases presented with hepatosplenomegaly and showed the presence of cytopenia on peripheral smears. Thomas et al. [23] interpreted that bone marrow involvement is common in storage disorders and they can present as haematological abnormalities. Bone marrow aspiration can help in confirming the diagnosis.

We reported two siblings with myelodysplastic syndrome, associated with familial monosomy 7. Their elder sister, who had associated monosomy 7 in 70% of her marrow cells, succumbed to AML at the age of eight years. The elder sibling was an eight-year-old male child who presented with a history of repeated episodes of fever, bleeding gums, petechial rash, and frequent platelet transfusions since he was four years old. The previous bone marrow examinations performed were normal. He was diagnosed as a case of chronic ITP. A clinical examination revealed pallor and splenomegaly, and a peripheral smear showed pancytopenia. Bone marrow aspirate was diluted with peripheral blood. Trephine biopsy revealed micronormoblastic and megaloblastic dyserythropoiesis with diffuse fibrosis of reticulin grade 2 to 3. No blasts were seen. Marked reductions in granulocyte series and small dysplastic megakaryocytes were seen in immunohistochemistry. Fluorescent-in-situ hybridization (FISH) revealed monosomy 7 in 90% of the marrow cells. A diagnosis of MDS was made. Over a period of four months, the child went into refractory cytopenia with the presence of blasts in the marrow. MDS transforming into AML was thought of. The child eventually expired.

The younger sibling, who was a four-year-old male child, was asymptomatic with normal counts on the complete haemogram report. His bone marrow examination revealed dyserythropoiesis and occasional dysplastic megakaryocytes. However, we did not receive the follow-up regarding the presence or absence of monosomy 7. Dysplasia in the bone marrow is one of the early indicators of MDS and AML. A future occurrence of mosaic monosomy 7 in the peripheral blood or bone marrow is not excluded by a normal karyotype. Regular follow-up was advised for the younger sibling for early detection of progression to AML.

A single case of JMML was reported in a female child of five months of age. She presented with fever, severe anaemia, leucocytosis, and massive splenomegaly. Her peripheral smear and bone marrow findings were similar to the findings seen in the studies described in the literature, with the peripheral smear showing monocytosis and circulating myeloid precursors and the bone marrow aspirate showing myelomonocytic hyperplasia, nucleated RBCs, and 8% abnormal cells. The trephine biopsy was inadequate to opine. Based on the clinical features, peripheral smear, and bone marrow aspirate findings, a diagnosis of JMML was suggested, and flow cytometry and cytogenetic studies were advised. Monosomy 7 was detected in this case, and BCR-ABL translocation was negative. HbF levels were raised. Thus, a diagnosis of JMML was made based on the criteria given in the WHO 2016 classification of myeloid neoplasms. JMML is a rare but lethal clonal myeloproliferative neoplasm of childhood. It accounts for <2-3% of all leukemias in children aged zero to 14 years. 75% of cases occur in children <3 years of age, with boys being affected twice as frequently as girls [24]. A bone marrow examination is often performed in suspected cases of JMML in order to exclude acute myelomonocytic leukemia.

Primary myelofibrosis in children is rare. Secondary myelofibrosis occurs for a variety of reasons, which range from systemic illnesses to malignancies. Only a few cases have been reported describing the relationship between vitamin D deficiency and rickets, despite its high prevalence. In the present study, a 1.5-year-old female child presented with severe anaemia, a cold, cough, low vitamin D levels, and features of full-blown rickets. A bone marrow aspirate yielded a dry tap and a bone marrow biopsy showed grade 3 myelofibrosis on reticulin stain. Venkatnarayan et al. [25] reported a single case of a 10-month-old male infant diagnosed as a case of vitamin D deficiency rickets, who presented with a delay in motor developmental delay, anemia, pallor, and abdominal distention. The aspirate yielded a dry tap while the biopsy showed features of myelofibrosis on reticulin stain, seen in our case as well.

TAR syndrome is a rare congenital condition. It presents as an amegakaryocytic thrombocytopenia with absent radii. The megakaryocytes in TAR syndrome are hypoplastic, that is, not developed properly. Thus, the normal maturation of megakaryocytes into platelets does not occur, leading to thrombocytopenia. During childhood, bleeding can be quite common due to coagulation dysfunction. As age increases, the symptoms improve and they may cause little or no problem in adulthood. After an extensive search in the literature, we did not come across the reason for it. Affected women, however, may have unusually heavy periods or menorrhagia. We received a single case (0.66%) of suspected TAR syndrome. The patient, who was a 16-year old female, presented with menorrhagia and thrombocytopenia with a platelet count of 65,000. Her haemoglobin and total leucocyte count were normal. An X-ray of the forearm showed an absent radius bone on both sides. A bone marrow examination revealed a normocellular marrow. Many monolobated megakaryocytes were seen. The erythroid series was micronormoblastic. Myeloid maturation was normal. There was a mild increase in eosinophils. There was no increase in blasts, plasma cells, histiocytes, or lymphocytes. There was no necrosis, fibrosis, or granuloma seen on trephine biopsy.

There were 15 cases where marrow was sent as a part of the management protocol and follow-up in children with acute leukaemia. This was to determine the marrow picture and blast count and compare them with the follow-up flow cytometry. Gupta et al. [26] stated that MRD (minimal residual disease) after initial therapy is integral for risk stratification in ALL and that remission remains defined by morphology, although MRD determines the depth of remission. The presence of <5% blasts on bone marrow defines remission on morphology. Our institute, where the present study was conducted, is a tertiary care centre with facilities for administering treatment to cancer patients. Thus, we got the opportunity to study the bone marrow of patients undergoing chemotherapy as a part of the morphological evaluation of marrows sent for residual disease. Fifteen bone marrow samples were received to evaluate morphological remission in cases of haematological malignancies involving the marrow. Of these, 53.3% (eight cases) were known cases of ALL, and 46.7% (seven cases) were known cases of AML. A bone marrow examination showed a normocellular marrow for 66.7% of cases and a hypocellular marrow for age for 33.3% of cases. No blasts were seen on the marrow in 14 cases. One patient, who was a 15-year-old male and a known case of AML, showed the presence of 8% blasts in the aspirate. Trephine biopsy of the same case was inadequate for opinion. Care was taken to count 200 to 500 cells so as to give the exact blast count. Bone marrow aspirate samples from all these cases were also sent for molecular remission status. However, the results could not be followed up.

A few limitations of the study noted were that it was a single-centre study and, hence, the results obtained may not apply to other regions. Our study was more focused on morphology; however, more details regarding flow cytometry/cytogenetics/certain laboratory parameters could have made the study more sturdy. Since many of our patients belonged to the medium to low income category, we presume that, for various reasons, they might not have been able to reach out to us, leading to incomplete follow-ups, which was beyond our control.

## Conclusions

A spectrum of lesions requiring bone marrow examination in the paediatric age group is of varied etiologies. Bone marrow examination directly helps to diagnose malignancies involving the marrow, infections, storage disorders, metabolic conditions (rickets), and relapse of haematological malignancies. Bone marrow aspiration and biopsy can offer contributions to molecular tests and flow cytometry for diagnosing remission/relapse. Vitamin D deficiency rickets is a disorder encountered in the paediatric age group. Myelofibrosis as a cause of severe anaemia in these patients should strongly be considered. Although uncommon, MDS is seen in children and can be familial. It usually presents with cytopenia and is associated with characteristic morphological features on bone marrow examination. Hemophagocytosis in the marrow can have a varied etiology, and its presence, which can be detected on bone marrow examination, can offer direction towards diagnosis. Bone marrow examination in children is thus useful in understanding the pathogenesis, establishing a tissue diagnosis, ruling out haematological malignancies and other causes of cytopenias, planning further lines of investigation and management, and knowing the response to therapy and prognosis.
